# 3-*O*-Acyl-epicatechins Increase Glucose Uptake Activity and GLUT4 Translocation through Activation of PI3K Signaling in Skeletal Muscle Cells

**DOI:** 10.3390/ijms160716288

**Published:** 2015-07-17

**Authors:** Manabu Ueda-Wakagi, Rie Mukai, Naoya Fuse, Yoshiyuki Mizushina, Hitoshi Ashida

**Affiliations:** 1 Department of Agrobioscience, Graduate School of Agricultural Science, Kobe University, Nada-ku, Kobe, Hyogo 657-8501, Japan; E-Mails: mana5998bu@affrc.go.jp (M.U.-W.); rmukai@tokushima-u.ac.jp (R.M.); hb03e708@gmail.com (N.F.); 2 National Agriculture and Food Research Organization, National Food Research Institute, Tsukuba, Ibaraki 305-8642, Japan; 3 Department of Food Science, Institute of Health Biosciences, University of Tokushima Graduate School, Kuramoto, Tokushima 770-8503, Japan; 4 Laboratory of Food and Nutritional Sciences, Faculty of Nutrition, Kobe Gakuin University, Nishi-ku, Kobe, Hyogo 651-2180, Japan; E-Mail: mizushina@shinshu-u.ac.jp; 5 Graduate School of Agriculture, Shinshu University, Minamiminowa-mura, Kamiina-gun, Nagano 399-4598, Japan

**Keywords:** acyl catechin, glucose transporter 4, skeletal muscle, insulin signaling pathway

## Abstract

Tea catechins promote glucose uptake in skeletal muscle cells. In this study, we investigated whether the addition of an acyl group to the C-3 position of catechins to generate 3-*O*-acyl-catechins promoted glucose uptake in L6 myotubes. 3-*O*-Myristoyl-(−)-epicatechin (EC-C14) and 3-*O*-palmitoyl-(−)-epicatechin (EC-C16) promoted glucose uptake and translocation of glucose transporter (GLUT) 4 in the cells. The effect of 3-*O*-acyl-(−)-epicatechins was stronger than that of (−)-epicatechin (EC), whereas neither 3-*O*-myristoyl-(+)-catechin (C-C14) nor 3-*O*-palmitoyl-(+)catechin (C-C16) promoted glucose uptake or GLUT4 translocation as well as (+)-catechin (C). We further investigated an affinity of catechins and 3-*O*-acyl-catechins to the lipid bilayer membrane by using surface plasma resonance analysis. Maximum binding amounts of EC-C16 and C-C16 to the lipid bilayer clearly increased compared with that of (−)-EC and (+)-C, respectively. We also examined the mechanism of GLUT4 translocation and found EC-C14 and EC-C16 induced the phosphorylation of PI3K, but did not affect phosphorylation of Akt or IR. In conclusion, the addition of an acyl group to the C-3 position of (−)-EC increases its affinity for the lipid bilayer membrane and promotes GLUT4 translocation through PI3K-dependent pathways in L6 myotubes.

## 1. Introduction

Catechins and their gallate esters are a class of polyphenols that includes the subclass known as flavan-3-ols. Catechins are natural active compounds contained in foods, such as tea, grapes, chocolate, apples, and berries [1]. Catechins have recently attracted a great deal of attention for their beneficial effects, including antioxidant [2], anti-inflammatory [3], anti-cancer [4], and anti-diabetic activities [5]. In our previous studies, it was demonstrated that green tea and tea catechins possessed a regulatory effect on glucose metabolism [6,7], especially, (−)-epigallocatechin gallate (EGCg), the major compound derived from green tea. EGCg promoted glucose uptake, along with glucose transporter (GLUT) 4 translocation in skeletal muscle cells [6].

GLUTs play an important role in the regulation of blood glucose levels. GLUT4 is specifically expressed in skeletal muscle and adipose tissue, and is mainly localized in intracellular storage vesicles. GLUT4 storage vesicles translocate to the plasma membrane with various stimuli and take up glucose to reduce postprandial hyperglycemia [8]. In skeletal muscle, the translocation is regulated by insulin and AMP-activated protein kinase signaling pathways [9]. In the insulin pathway, binding of insulin activates the tyrosine kinase activity of its receptor, which phosphorylates insulin receptor substrate-1 (IRS-1) followed by phosphorylation of the p85 regulatory subunit of phosphatidylinositol 3ʹ-kinase (PI3K). Activated PI3K induces phosphorylation of Akt to regulate GLUT4 translocation. Thus, GLUT4 translocation is regulated by a complex cascade of multiple protein kinases, and finally appears on the plasma membrane.

Many researchers have investigated the bioactivities of natural catechin derivatives and synthesized catechins by comparing them to endogenous catechins. For example, 3-*O*-methylated epicatechin gallate and 3-*O*-metylated epigallocatechin gallate, components in the “Benifuuki” green tea cultivar (*Camellia sinensis* L.), exhibited greater inhibition of histamine release from murine bone marrow mast cells than (−)-EC gallate and EGCg, respectively [10]. Synthesized 3-*O*-alkyl (+)-catechin exhibited greater antimicrobial activity than (+)-C due to disruption of the liposome membrane [11,12]. (−)-EC conjugated with fatty acid strongly inhibited DNA polymerase activity and angiogenesis in human endothelial cells [13]. These reports suggest natural catechin derivatives and synthesized catechin derivatives increase the lipophilicity, which may contribute to the increase their bioactivities. Our previous study showed that tea catechins, except for (+)-C and (−)-catechin gallate, significantly increased glucose uptake activity in skeletal muscle cells [6]. In this study, therefore, we investigated whether synthesized 3-*O*-acyl-catechins increase glucose uptake activity in L6 myotubes. We also investigated an affinity of 3-*O*-acyl-catechins for lipid bilayer membrane using surface plasmon resonance (SPR) analysis.

## 2. Results

### 2.1. 3-O-Acyl-(−)-epicatechins Promote Glucose Uptake in L6 Myotubes

**Figure 1:**
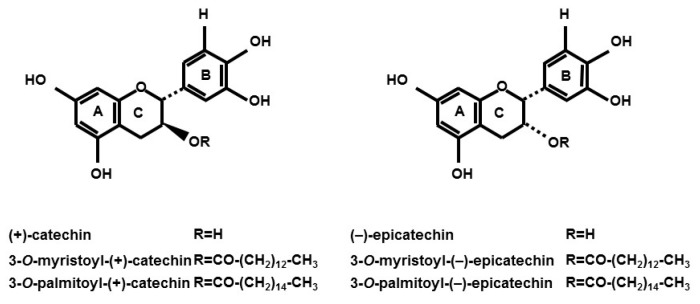
The chemical structures of 3-*O*-acyl-catechins.

**Figure 2:**
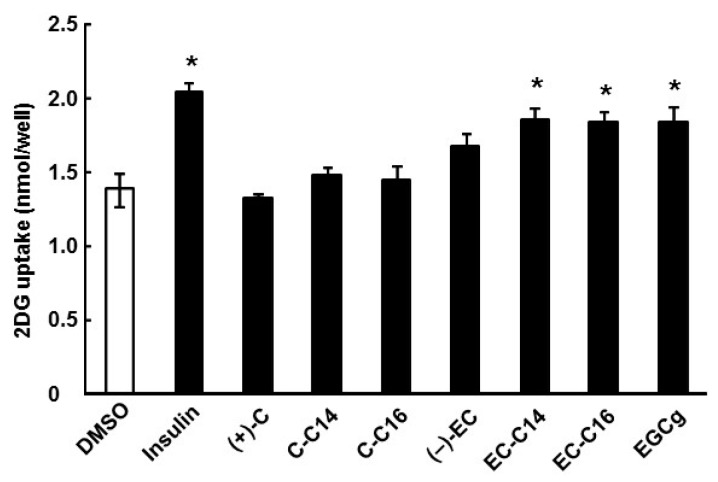
The effects of 3-*O*-acyl-catechins on glucose uptake activity in L6 myotubes. Differentiated L6 cells were incubated with 100 nM catechins and 3-*O*-acyl-catechins for 15 min. Glucose uptake activity was measured using [^3^H]-2DG as described in the Experimental Section. Data are shown as the mean ± SE (*n* = 3). * *p* < 0.05 *vs.* DMSO-treated control cells (Dunnett multiple comparison test).

Structures of catechins used are shown in Figure 1. In this study, we first investigated whether 3-*O*-acyl-catechins promote glucose uptake activity in L6 myotubes. As shown in Figure 2, 3-*O*-myristoyl-(−)-epicatechin (EC-C14), 3-*O*-palmitoyl-(−)-epicatechin (EC-C16), and EGCg significantly increased glucose uptake activity by 1.33-, 1.34-, and 1.33-fold, respectively, compared with DMSO-treated cells. The effects of 3-*O*-acyl-(−)-epicatechins were stronger than those of the original compound, (−)-EC. Conversely, 3-*O*-myristoyl-(+)-catechin (C-C14) and 3-*O*-palmitoyl-(+)-catechin (C-C16) did not increase glucose uptake activity as effectively as (+)-C. Moreover, the addition of the acyl group (carbon chain lengths from 6 to 18) to the C-3 position of (+)-C also did not affect the glucose uptake activity (Figure S1A). Catechins and 3-*O*-acyl-catechins used in this study did not show any cytotoxicity under the experimental conditions (Table S1). These results indicate that the addition of an acyl group to the C-3 position of (−)-EC improves glucose uptake activity in L6 myotubes.

### 2.2. 3-O-Acyl-(−)-epicatechins Promote GLUT4 Translocation in L6 Myotubes

**Figure 3:**
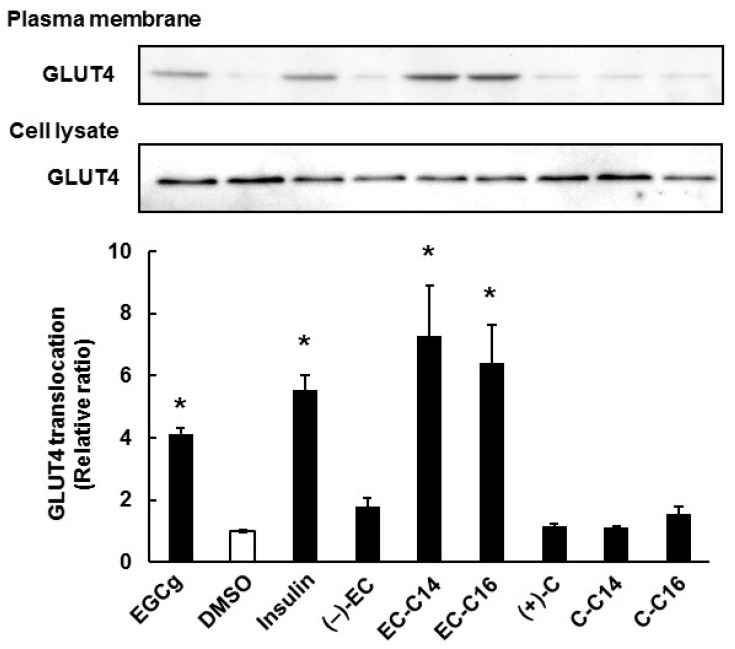
The effect of 3-*O*-acyl-catechins on GLUT4 translocation in L6 myotubes. Differentiated L6 cells were incubated with catechins and 3-*O*-acyl-catechins at 100 nM for 15 min. GLUT4 in the plasma membrane and the cell lysate were detected by Western blot analysis. Band density was determined by ImageJ analysis software. Data are shown as the mean ± SE (*n* = 3). * *p* < 0.05 *vs.* DMSO-treated control cells by a Dunnett multiple comparison test.

**Figure 4:**
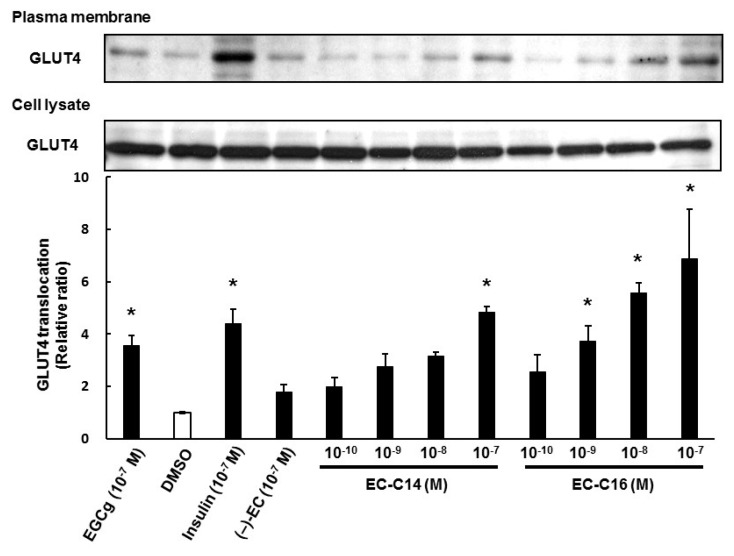
The dose-dependent effects of 3-*O*-acyl-(−)-epicatechins on GLUT4 translocation in L6 myotubes. Differentiated L6 cells were incubated with 3-*O*-acyl-catechins at the indicated concentrations for 15 min. GLUT4 in the plasma membrane and the cell lysate were detected by Western blot analysis. Band density was determined by ImageJ analysis software. Data are shown as the mean ± SE (*n* = 3). * *p* < 0.05 *vs.* DMSO-treated control cells by a Dunnett multiple comparison test.

**Figure 5:**
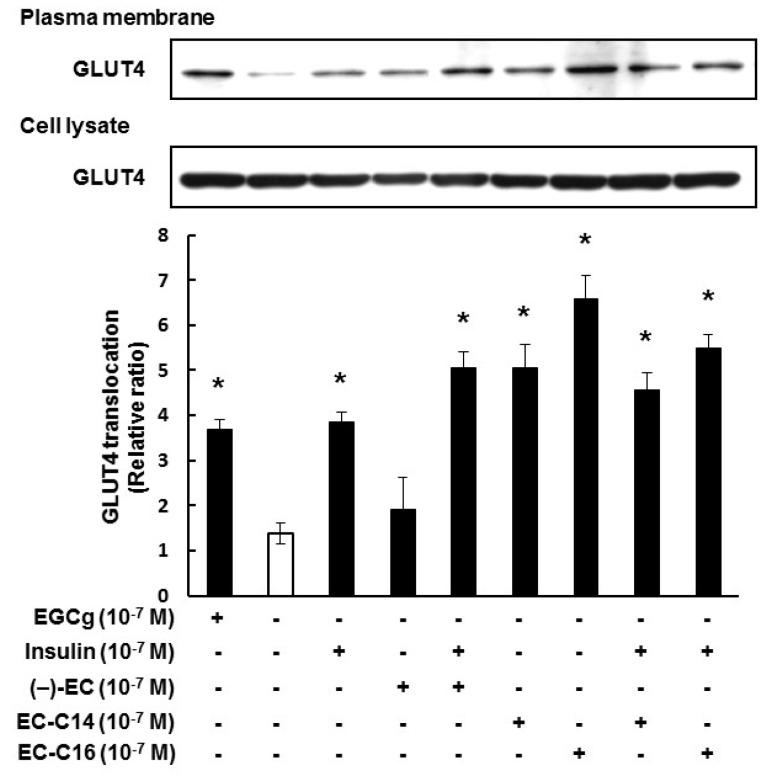
The effects of 3-*O*-acyl-(−)-epicatechins on GLUT4 translocation in the presence of insulin in L6 myotubes. Differentiated L6 cells were incubated with 3-*O*-acyl-(−)-epicatechins at 100 nM in the presence and absence of insulin at 100 nM for 15 min. GLUT4 in the plasma membrane and the cell lysate were detected by Western blot analysis. Band density was determined by ImageJ analysis software. Data are shown as the mean ± SE (*n* = 3). * *p* < 0.05 *vs.* DMSO-treated control cells by a Dunnett multiple comparison test.

We next investigated the effects of 3-*O*-acyl-catechins on the translocation of GLUT4 to the plasma membrane in L6 myotubes. As shown in Figure 3, EC-C14 and EC-C16 promoted GLUT4 translocation in L6 myotubes, whereas C-C14 and C-C16 did not (also see Figure S1B). These results were almost the same trend as their glucose uptake activities. As shown Figure 4, EC-C14 and EC-C16 promoted GLUT4 translocation in a dose-dependent manner, and a significant increase was observed in L6 myotubes at a concentration of 100 nM. These results indicated that 3-*O*-acyl-(−)-epicatechins increase glucose uptake activity accompanied with GLUT4 translocation in L6 myotubes. Our previous study demonstrated that gallate-type catechins decreased insulin-induced glucose uptake activity in 3T3-L1 adipocytes, whereas non-gallate-type catechins did not [7]. Therefore, we investigated the effects of 3-*O*-(−)-acyl-epicatechins on GLUT4 translocation in the presence and absence of insulin in L6 myotubes (Figure 5). EC-C14 and EC-C16 at 100 nM promoted GLUT4 translocation in the presence and absence of insulin, but neither competitive nor additive effects between 3-*O*-acyl-(−)-epicatechins and insulin were observed. This result indicated that the mechanism of 3-*O*-acyl-(−)-epicatechins on GLUT4 translocation might be, at least in part, different from that of insulin.

### 2.3. 3-O-Acyl-(−)-epicatechins Promote GLUT4 Translocation in L6 Myotubes

**Figure 6:**
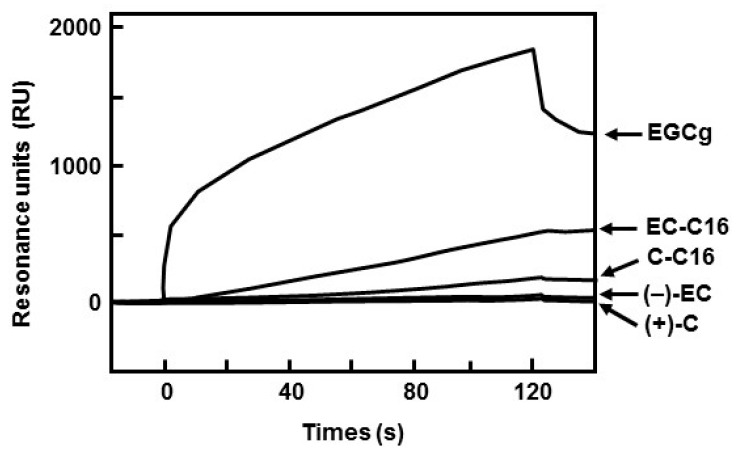
Binding between 3-*O*-acyl-catechins and lipid bilayer membrane. The sensorgrams indicated binding after injection of 130 μL of 10 μM catechins and their derivatives at a flow rate of 65 μL/min.

It was reported that bioactivities of catechins depended on their affinity for lipid bilayer membrane [14,15]. Therefore, we estimated the binding affinity of 3-*O*-acyl-catechins to lipid bilayer membrane using SPR analysis. Acyl group clearly increased the maximum binding amount of both (−)-EC and (+)-C to the lipid bilayer membrane (Figure 6). As to affinity between catehins and the membrane, the dissociation constant (*K*_D_) of (+)-C, (−)-EC, EGCg, C-C16, and EC-C16 were determined as 5.84 × 10^−4^, 1.39 × 10^−8^, 8.26 × 10^−10^, 1.63 × 10^−7^ and 7.87 × 10^−8^ M, respectively (Table 1). This result showed that the affinity of C-C16 for lipid bilayer membrane was stronger than that of (+)-C, whereas addition of acyl group to the C-3 position of (−)-EC did not affect the affinity. From these results, we suggest that addition of an acyl group to C-3 position of catechins has an advantage in binding of the compound to the cellular membrane, and that combination of the acyl group and geometrical structure of catechins is responsible for degree of affinity to the membrane.

**Table 1:** Affinities for the interaction between catechins and lipid bilayer.

Catechin	*K*_A_ (1/M)	*K*_D_ (M)
(+)-C	1.71 × 10^3^	5.84 × 10^−4^
(−)-EC	7.19 × 10^7^	1.39 × 10^−8^
EGCg	1.21 × 10^9^	8.26 × 10^−1^°
C-C16	6.12 × 10^6^	1.63 × 10^−7^
EC-C16	1.27 × 10^7^	7.87 × 10^−8^

### 2.4. 3-O-Acyl-(−)-epicatechins Promote GLUT4 Translocation through a PI3K-Dependent and Insulin-Independent Pathway

**Figure 7:**
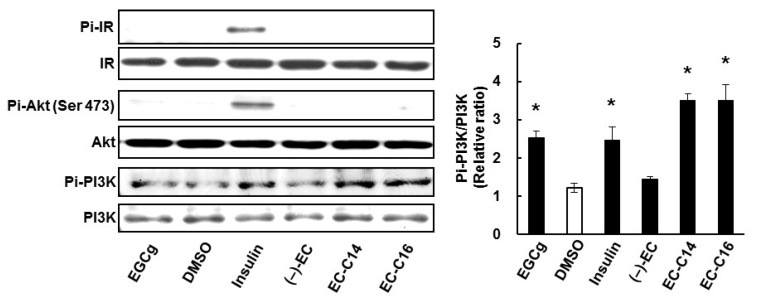
The effects of 3-*O*-acyl-(−)-epicatechins on GLUT4 translocation on insulin signaling pathway in L6 myotubes. Differentiated L6 cells were incubated with 3-*O*-acyl-(−)-epicatechins at 100 nM for 15 min. The phosphorylation and expression of IR, Akt, and PI3K were detected by Western blot analysis. Band density was determined by ImageJ analysis software. Data are shown as the mean ± SE (*n* = 3). * *p* < 0.05 *vs.* DMSO-treated control cells by a Dunnett multiple comparison test.

Because insulin-induced translocation of GLUT4 requires the activation of several proteins in the insulin signaling pathway, such as IR, PI3K, and Akt [8], we investigated whether 3-*O*-acyl-(−)-epicatechins activate the insulin signaling pathway in L6 myotubes. As shown in Figure 7, insulin promoted phosphorylation of IR and Akt, but 3-*O*-acyl-(−)-epicatechins did not affect this phosphorylation. However, 3-*O*-acyl-(−)-epicatechins promoted phosphorylation of PI3K in the same manner as insulin and EGCg. It was confirmed that expression levels of these proteins remained unchanged after treatment with either 3-*O*-acyl-(−)-epicatechins or insulin. 3-*O*-Acyl-(−)-epicatechins therefore increases glucose uptake activity and GLUT4 translocation through activation of PI3K signaling in L6 myotubes, but the underlying mechanism of this action is different from that of insulin.

## 3. Discussion

Our previous study showed that tea catechins, especially EGCg, promoted glucose uptake in L6 myotubes [4]. In this study, we found that the addition of acyl group to the C-3 position of (−)-EC increased the glucose uptake activity. This is the first report of acyl catechin derivatives influencing glucose uptake activity in skeletal muscle cells. Our findings support previous results that catechin derivatives increased their bioactivity compared with the original compound(s): Matsubara *et al.* [13] reported that *O*-acyl derivatives of epicatechin strongly inhibited DNA polymerase activity, growth of HL-60 cancer cells and angiogenesis in human endothelial cell, and EC-C16 had the strongest inhibition. Park *et al.* [12] reported that 3-*O*-acyl and alkyl-(−)-epicatechin derivatives strongly inhibited the growth of cancer cell lines (PC3, SKOV3, U373MG). Stapleton *et al.* [16] reported that the addition of an acyl group to the C-3 position of (+)-C and (−)-EC significantly increased the bactericidal activity. Thus, acyl catechins are attractive compounds with beneficial functions including anti-hyperglycemic activity.

In this study, we found that the addition of acyl group to the C-3 position of (−)-EC increased the affinity for lipid bilayers membrane (Figure 6 and Table 1). The affinity for lipid bilayer membrane was correlated with glucose uptake activity in L6 myotubes, which supports previous reports that bioactivities of catechin were associated with the affinity for lipid bilayer membrane. For example, Kajiya *et al.* [15] reported that the order of partition coefficient of (+)-C derivatives was closely correlated with the amount of derivatives incorporated into the lipid bilayers, attributed to the bactericidal activity. From these results, the affinity of catechin derivatives for lipid bilayer membrane is important factor for glucose uptake activity in L6 myotubes. In this study, we also found that 3-*O*-acyl-(+)-catechin (chain lengths of C6–C18) did not promote glucose uptake activity and GLUT4 translocation as well as (+)-C in L6 myotubes (Figure 2, Figure 3 and Figure S1). These results suggest that the chemical structure of catechins, in particular the *cis*-type structure, is also an important factor for glucose uptake activity. We showed similar results where (−)-EC gallate increased glucose uptake activity compared with (−)-EC, whereas (−)-catechin gallate did not increase activity as well as (+)-C [6]. Uekusa *et al.* [17,18] reported that the B ring and galloyl moiety of *cis*-type catechins were located near the trimethylammonium group (γ position) of phospholipids, contributing to their affinity for lipid bilayer membrane. From our findings and these previous results, we suggest that the B ring and the C-3 position of catechin derivatives are important to glucose uptake activity.

Regarding the mechanism of GLUT4 translocation, 3-*O*-acyl-(−)-epicatechins induced phosphorylation of PI3K but not IR in L6 myotubes in the same manner as EGCg (Figure 7). Although the target molecule for 3-*O*-acyl-(−)-epicatechins was not identified in this study, we hypothesize that these compounds affect receptor type tyrosine kinases in the membrane of skeletal muscle cells, and that their affinity for lipid bilayer membrane may be involved in the observed glucose uptake activity. It has been reported that (−)-epicatechin gallate, but not (−)-EC, inhibited tyrosine phosphorylation of vascular endothelial growth factor receptor [14]. However, there is currently no report describing the target molecules of EGCg responsible for mediating its anti-hyperglycemic effects, including GLUT4 translocation. One possible candidate is ErbB3, because this receptor type tyrosine kinase is involved in GLUT4 translocation in L6 myotubes by activating PI3K and PKCλ/ξ [19]. Further studies are, therefore, needed to confirm the molecular target for 3-*O*-acyl-(−)-epicatechins.

## 4. Experimental Section

### 4.1. Chemicals and Reagents

Catechins were obtained from Kurita Water Industries Ltd. (Tokyo, Japan). The acylated catechin and epicatechin derivatives (3-*O*-acyl-flavan-3-ols), which are conjugated fatty acids, were chemically synthesized as described previously [13,20]. To measure glucose uptake, [1, 2-^3^H]-2-deoxy-d-glucose (2-DG) was purchased from American Radiolabeled Chemicals, Inc. (St. Louis, MO, USA). For Western blotting analysis, anti-GLUT4 goat IgG, anti-phospho-PI3K (Tyr 508) goat IgG, anti-mouse IgG, anti-goat IgG, and anti-rabbit IgG antibodies were purchased from Santa Cruz Biotechnology Inc. (Santa Cruz, CA, USA), anti-PY20 mouse IgG and anti-PI3K goat IgG antibodies from BD Transduction Laboratories Ltd. (San Diego, CA, USA), and anti-IR rabbit IgG, anti-phospho-Akt (Ser473) rabbit IgG and anti-β-actin mouse IgG antibodies from Sigma Chemical Co. (St. Louis, MO, USA). Protease and phosphatase inhibitor cocktails were purchased from Roche Diagnostics K.K. (Tokyo, Japan). All other reagents used were of the highest grade available in commercial products.

### 4.2. Cell Culture and Glucose Uptake Assay

Culture of L6 myoblasts and differentiation to myotubes were performed as previously described [21]. Briefly, L6 myoblasts were cultured and differentiated on a 24-well plate and serum-starved for 18 h in MEM containing 0.2% BSA at 37 °C. The cells were incubated with (+)-C, (−)-EC, EGCg, and 3-*O*-acyl-catechins at 100 nM in Krebs-Ringer phosphate-HEPES buffer (KRH; 50 mM HEPES, pH 7.4, 137 mM NaCl, 4.8 mM KCl, 1.85 mM CaCl_2_, and 1.3 mM MgSO_4_) for 15 min. Then, the cells were incubated with [^3^H]-2-DG at a final concentration of 6.5 mM (0.5 μCi) and incubated for 5 min at 37 °C. For positive and negative controls, the cells were treated with 100 nM insulin or DMSO (final 0.1%) for 15 min. The uptake of [^3^H]-2-DG was terminated by immediately washing the myotubes with ice-cold KRH four times. Non-specific uptake was measured in the presence of 20 μM cytochalasin B, a glucose transport inhibitor. After the cells were solubilized with 50 mM NaOH, incorporated radioactivity was measured by liquid scintillation counting using a scintillation cocktail.

### 4.3. Western Blot Analysis and Immunoprecipitation

Differentiated L6 myotubes were treated with 3-*O*-acyl-catechins at the concentrations indicated in each figure for 15 min. As positive and negative controls, cells were treated with 100 nM insulin and DMSO (final 0.1%) for 15 min. The plasma membrane fraction and cell lysate were prepared and subjected to SDS-PAGE followed by Western blot analysis to detect GLUT4 translocation and the expression and phosphorylation of proteins related to the translocation [21]. Primary and secondary antibodies were diluted 1:10,000 and 1:20,000, respectively, in Can Get Signal (Toyobo Co., Ltd., Osaka, Japan).

To detect phosphorylation of IR, 200 μg of cell lysate was incubated with 5 μL of 50% protein A/G plus-agarose suspension (Santa Cruz Biotechnology Inc.) for 1 h at 4 °C and centrifuged to remove non-specific proteins. The mixture was centrifuged at 1000× *g* for 5 min, and the supernatant was incubated with 5 μL of anti-PY20 overnight at 4 °C. New protein A/G plus-agarose suspension (5 μL) was added to this mixture and incubated for 1 h at 4 °C. After washing the agarose resin four times with ice-cold RIPA buffer (10 mM Tris, pH 8.0, 150 mM NaCl, 1.0% NP-40, 0.5% sodium deoxycholate, 0.1% sodium dodecyl sulfate (SDS), 0.5 mM dithiothreitol (DTT), and protease and phosphatase inhibitor cocktails) under the same centrifugation conditions as described above. The precipitated protein A/G plus-agarose resins were subjected to SDS-PAGE followed by Western blotting to detect phosphorylation of IR.

### 4.4. Surface Plasmon Resonance (SPR) Analysis

To prepare small unilamellar vesicles, phosphatidylcholine was dissolved in ethanol-free chloroform to approximately 10 mg/mL and vacuum dried using a rotary evaporator. The dried lipid was gently resuspended in 120 mM phosphate buffered saline (PBS) pH 7.4 to a lipid concentration of 20 mM and shaken for 5 min. This suspension was further diluted with PBS to the concentration of 2 mM, and sonicated in a bath sonicator for 2 h.

To detect interaction between 3-*O*-acyl-catechins and lipid bilayer membrane, SPR analysis was performed using a Biacore 3000 (GE Healthcare, Piscataway, NJ, USA) equipped with an L1 sensor chip. The sensor chip was cleaned by an injection of the nonionic detergent, 40 mM *n*-octyl β-d-glucoside, at a flow rate of 10 μL/min for 5 min. Small unilamellar vesicles at 0.5 mM were then applied to the sensor chip surface at the low flow rate (2 μL/min) for 1 min. To remove any multilamellar structures from the lipid surface, 50 mM NaOH was injected at the flow rate (2 μL/min), which resulted in a stable baseline corresponding to the lipid bilayer linked to the chip surface. As the negative control, BSA (100 μg/mL in PBS) was injected to confirm complete coverage of the nonspecific binding sites. The bilayer linked to the chip surface was then used as a model cell membrane surface to study binding of 3-*O*-acyl-catechins to the membrane. (+)-C, (−)-EC, EGCg, and 3-*O*-palmitoyl-catechins (C-C16 and EC-C16) at 10 μM in PBS containing 1% DMSO were injected to the chip at the flow rate at 65 μL/min for 2 min. All reactions were carried out at 30 °C. Affinity constants were calculated using BIAevaluation 4.1 software by globally fitting the association.

### 4.5. Statistical Analysis

Statistical analyses were performed with factorial analysis of variance followed by a Dunnett multiple comparison test (Figure 2, Figure 3, Figure 4, Figure 5 and Figure 7). The level of significance was defined as *p* < 0.05.

## 5. Conclusions

In this study, we found that addition of an acyl group to the C-3 position of (−)-EC not only increased glucose uptake activity, but also increased the affinity of the molecule for the lipid bilayer membrane compared with (−)-EC in L6 myotubes. Conversely, the addition of the acyl group to the C-3 position of (+)-C also increased the affinity of the molecule for the lipid bilayer membrane, but it did not affect glucose uptake activity in the cells. These findings could contribute to the development and synthesis of catechin derivatives with the potential to prevent and/or improve diabetes mellitus and hyperglycemia, because glucose uptake activity in skeletal muscle mainly regulates blood glucose level at postprandial period.

## Supplementary Materials

Supplementary materials can be found at http://www.mdpi.com/1422-0067/16/07/16288/s1.
