# Supplementary material for: 3-O-Acyl-epicatechins Increase Glucose Uptake Activity and GLUT4 Translocation through Activation of PI3K Signaling in Skeletal Muscle Cells
Source: Int J Mol Sci. 2015 Jul 17;16(7):16288–99. doi: 10.3390/ijms160716288 (PMC4519950; doi:10.3390/ijms160716288)
Supplement: Supplementary file 1 [file ijms-16-16288-s001.pdf]

Supplementary Information

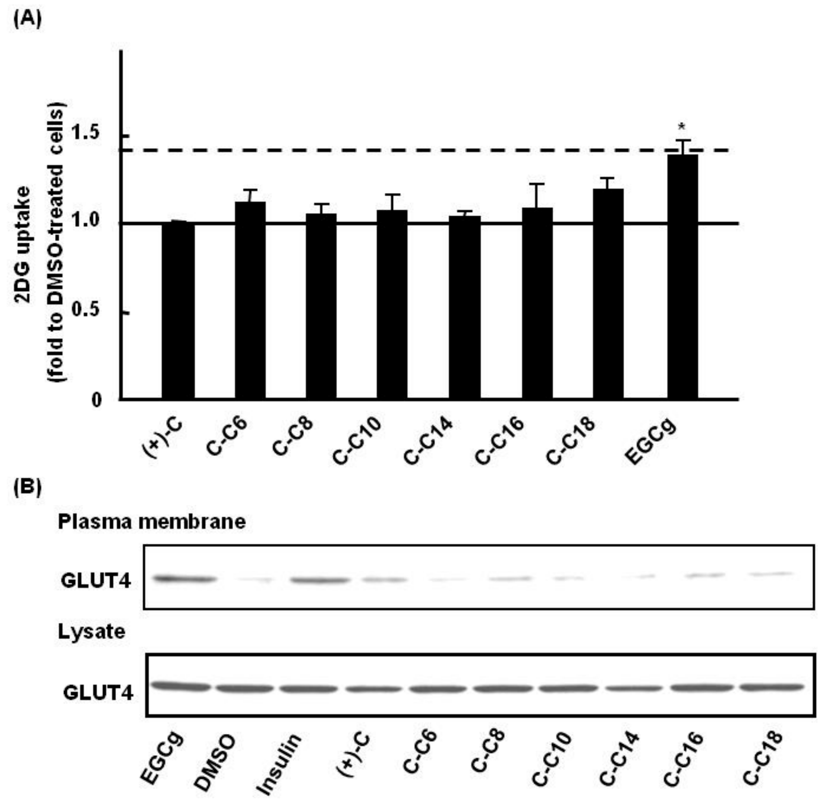

Figure S1. Effects of 3-*O*-acylcatechins on glucose uptake activity and GLUT4 translocation in L6 myotubes. Differentiated L6 cells were incubated with 100 nM catechins and 3-*O*-acyl-catechins for 15 min. (**A**) Glucose uptake activity was measured using [^{3}H]-2DG as described in the Experimental section. Data are shown as the mean ± SE (n = 3). * $p < 0.05$ *vs.* DMSO-treated control cells (Dunnett multiple comparison test); (**B**) GLUT4 in the plasma membrane and the cell lysate were detected by Western blot analysis.

Table S1. Effects of acyl-catechins on cell viability of L6 myotubes.

Catechin (M)	Cell Viability (% of DMSO-Treated Cells)		
	10^{-7}	10^{-6}	10^{-6}
(+)-C	100 ± 1.95	97 ± 2.86	88 ± 3.66
C-C6	96 ± 2.35	100 ± 4.53	75 ± 2.17 *
C-C8	99 ± 2.02	94 ± 1.49	49 ± 2.41 *
C-C10	96 ± 2.70	90 ± 1.80	38 ± 1.19 *
C-C14	89 ± 3.46	88 ± 2.78	42 ± 7.67 *
C-C16	109 ± 3.07	97 ± 2.78	77 ± 1.62 *
C-C18	106 ± 2.05	96 ± 3.67	57 ± 0.91 *
(−)-EC	98 ± 2.80	96 ± 3.21	101 ± 3.23
EC-C14	101 ± 6.17	94 ± 2.03	76 ± 5.49 *
EC-C16	103 ± 1.73	98 ± 2.67	87 ± 1.86 *

Differentiated L6 cells were incubated with the indicated concentration of catechins for 24 h. Cell viability was measured by WST-1 assay. Data are represented as % of control cells treated with 0.1% DMSO and expressed as the means ± SE (n = 3). Asterisks indicate significant differences from the DMSO-treated control cells, $p < 0.05$ by Student's t-test.
